# Editorial: Psychological Factors in Physical Education and Sport

**DOI:** 10.3389/fpsyg.2021.826291

**Published:** 2022-01-13

**Authors:** Marianna Alesi, Manuel Gómez-López, Carla Chicau Borrego

**Affiliations:** ^1^Department of Psychology, Educational Sciences, and Human Movement, University of Palermo, Palermo, Italy; ^2^Department of Physical Activity and Sport, Faculty of Sports Sciences, University of Murcia, Murcia, Spain; ^3^Sport Sciences School of Rio Maior, Polytechnic Institute of Santarém, Research Center in Life Quality, Rio Maior, Portugal

**Keywords:** motivation, education, sport, physical education, psychological well-being

During the last decades, human development has been extensively investigated by a whole approach centered on a widespread description of growth. This multidimensional perspective underlies the close interaction between the individual and context, focusing on the body, person, and society, as the main components describing personal functioning, both in health and disability.

Moreover, it is becoming increasingly a shared opinion that an inactive lifestyle is a risk factor for significant rates of psychosocial impairments, onset, or exacerbation of medical diseases, and welfare assistance, with a consequent increase in health costs.

Following this perspective, the systematic and regular practice of physical activity (PA) is considered a significant factor that positively affects the physical and mental health of participants. PA acts as a pivotal factor contributing to well-being and health across the entire lifespan, from childhood to old age, and in different contexts ranging from school with physical education classes to specialized contexts such as gyms and competitive sports.

So, in all these contexts, such as sports and physical education classes, awareness, knowledge, and management of psychological variables such as attitude (Cruz et al.), attention, self-confidence, stress control, anxiety, motivation, cohesion, self-control, emotional self-regulation, and interpersonal skills can influence compliance to PA and sports performance.

Regarding the younger ages, in school and after-school contexts, the psychological benefits of PA are primarily recognized for cognitive, emotional, and social development. PA can influence global development through multiple pathways by creating an “enriched environment” that demands multiple cognitive, motivational, and emotional functions, thus challenging and enhancing them (Gentile et al.). These pathways consist of exciting and enjoyable programs, opportunities for social interaction, cooperation with peers, sharing leisure experiences, enhancing the sense of belonging to a group, increasing the sense of mastery, and personal competence. Moreover, a growing amount of research pointed to the effectiveness of motor programs and moderate to vigorous aerobic exercise programs to improve children's executive functions, given the close interplay between cognitive and motor domains (Gentile et al.).

Hybrid and mixed education programs, based on teaching personal and social responsibility and gamification strategies, are also an example of the benefits, in cardiorespiratory fitness, agility, speed, APA-weekdays and APA-weekends, and reducing the sedentary time. Again, the development of Specialist Sports Programs demonstrates a positive influence on students' engagement with school, and that this engagement had a positive impact on their academic achievement (Melero-Cañas et al.).

Sport is an emotional experience. Studies have shown that high emotional intelligence (EI) is associated with better sports performance, though different aspects of sports experience and their relationship with EI are still unclear. This study examined the possible relationships between sports experience and EI dimensions of undergraduate athletes (Rodriguez-Romo et al.).

The use of technology, in the form of individualized heart rate feedback during running, was an indicator that should be implemented in regular PE classes systematically and repeatedly to create a controllable and attainable situation that allows students to actively adjust their own behavior to achieve appealing and realistic goals, as it apparently increased the motivation of participants to run and to enjoy running at higher levels of exertion (Stöckel and Grimm). Also, the smartphone management of smartphone app-use patterns is important for the performance of professional golfers (Lee et al.).

Sports activities trigger not only essential technical elements but motor and cognitive growth. For example, during a football match players need to respond quickly and correctly to the actions enacted by teammates and opponents during the game, monitor the match conditions, and play strategies. Therefore, football movements require high-level cognitive processes to perceive and analyze changing play situations, decision-making by choosing tactical strategies, and performing technical and kinetic abilities (Zhou).

Nevertheless, as concerns the sports context, research emphasized psychological components as crucial factors to increase athlete performance (Samełko et al.; Martínez et al.). Sports success is the result of the interplay among technical skills and an adaptive motivational profile. This profile is multifaceted and derives from positive self-esteem and sport self-efficacy, adequate perception of competence, causal attribution to the effort, task-oriented goals, and persistence in the face of difficulties. These components predispose the athletes for the maximum use of sports skills by maximizing their sports efficiency. Criteria to mark sports success includes more than athletes' personal characteristics (sport skills and motivational profile), such as the social environment and the motivational climate created by coaches. In detail, a mastery-oriented motivational climate reinforces the perception of performance as a result of task mastery and outlines skills as potentials that can be enhanced by personal effort. On the other hand, an ego-oriented motivational climate reinforces the competition and rivalry with team members by producing anxiety and decreasing enjoyment and satisfaction within the sports environment.

Furthermore, it has also been confirmed that a highly competitive context can generate fear of failure and feelings of shame, causing some degree of insecurity, stress or burnout, and avoidance behaviors in athletes and students. All these factors, in turn, negatively affect well-being, interpersonal behavior, and performance (González-Hernández et al.).

The Pandemic has had an impact on sport, and higher physical activity levels may help buffer the negative psychological consequences of Coronavirus Disease (Antunes et al.).

Based on these theoretical premises, this Research Topic aimed to address essential questions and collect the most recent research on factors influencing physical and psychological well-being and adherence to PA in the context of physical education classes and sport.

## Author Contributions

All authors listed have made a substantial, direct, and intellectual contribution to the work and approved it for publication.

## Conflict of Interest

The authors declare that the research was conducted in the absence of any commercial or financial relationships that could be construed as a potential conflict of interest.

## Publisher's Note

All claims expressed in this article are solely those of the authors and do not necessarily represent those of their affiliated organizations, or those of the publisher, the editors and the reviewers. Any product that may be evaluated in this article, or claim that may be made by its manufacturer, is not guaranteed or endorsed by the publisher.

